# Transverse Relaxation Rate of Cyst Fluid Can Predict Malignant Transformation of Ovarian Endometriosis

**DOI:** 10.2463/mrms.mp.2016-0028

**Published:** 2016-09-16

**Authors:** Chiharu Yoshimoto, Junko Takahama, Takuya Iwabuchi, Masato Uchikoshi, Hiroshi Shigetomi, Hiroshi Kobayashi

**Affiliations:** 1 Department of Obstetrics and Gynecology, Nara Medical University, 840 Shijo-cho, Kashihara 634-8522, Japan; 2 Department of Radiology, Nara Medical University, Nara, Japan; 3 Department of Research and Development, Metallogenics Co., Ltd., Chiba, Japan; 4 Siemens Japan K.K., Tokyo, Japan

**Keywords:** endometriosis, carcinogenesis, magnetic resonance imaging, relaxometry, iron

## Abstract

**Purpose:**

Heme and iron accumulation due to repeated hemorrhage in endometriosis may contribute to a pivotal role in carcinogenesis. We evaluate the clinical application of MR relaxometry in a series of ovarian endometriosis (OE) and endometriosis-associated ovarian cancer (EAOC).

**Materials and Methods:**

A prospective study of diagnostic accuracy was conducted among 82 patients (67 OE and 15 EAOC) to compare MR relaxometry and biochemical measurement of cyst fluid total iron concentration. Transverse relaxation rate R2 value was determined using a single-voxel, multi-echo MR sequence (HISTO) by a 3T-MR system. Phantom experiments were also performed to assess the correlation between the *ex vivo* R2 values and total iron concentrations.

**Results:**

Both the results of phantom experiments and *in vivo* human data confirmed that *in vivo* R2 values were highly correlated with total iron concentrations. Compared to OE, EAOC exhibit decreased *in vivo* R2 values and total iron levels, regardless of their age, menopausal status and cyst size. The use of *in vivo* R2 values retained excellent accuracy in distinguishing EAOC versus OE (sensitivity and specificity: 86% and 94%).

**Conclusions:**

We have demonstrated that MR relaxometry provides a noninvasive predictive tool to discriminate between EAOC and OE.

## Introduction

Endometriosis is one of the most common diseases in women of reproductive age.^1^ Symptoms of dysmenorrhea, chronic pelvic pain and infertility negatively affect patients’ quality of life.^1^ The ovarian cancer risk was found to be elevated among women with ovarian endometriosis (OE) (odd ratio, 3.43–8.95).^2–5^ Heme and iron accumulation due to repeated hemorrhage in endometriosis may contribute to carcinogenesis by a range of possible mechanisms, including oxidative damage and chronic inflammation.^6,7^ There is also evidence of an epidemiologic link between iron overload and various types of human carcinoma, including malignant mesothelioma, renal cell carcinoma, hepatocellular carcinoma, and endometriosis-associated ovarian cancer (EAOC).^8^ This evidence was also supported by a laboratory-based experimental study showing that the iron-induced persistent oxidative stress has been associated with carcinogenesis.^9^ A recent study showed that, compared to OE, EAOC exhibited decreased total iron concentrations in cyst fluids.^10^ This data led us to speculate that cyst fluid iron levels could be a marker for EAOC.

MR imaging is a widely used in the assessment of endometriosis.^11^ MR relaxometry is used in chemical and biological sciences as an analytical method that enables the identification and quantification of metabolites and the chemical contents in biological fluids and tissues samples.^12^ MR sequences such as multi-echo ${\text{T}}_{2}^{*}$ corrected single voxel spectroscopy is suited to analyze a small volume of *in vivo* tissue while avoiding any sample manipulation. This type of imaging has been used in clinical studies to estimate tissue iron deposition in the brain,^13–17^ liver^18–23^ and other tissues.^24–26^

Our objective was to investigate 1) whether the MR relaxometry-based parameter applied in OE and EAOC shows a significant correlation with the in vitro iron concentrations, and 2) whether this method provides the differential diagnosis of EAOC from OE.

## Materials and Methods

### Subjects and study design

This is a single-center, non-randomized, prospective cohort study to assess the accuracy of MR relaxometry for the estimation of cyst fluid total iron concentration and accurate diagnosis of malignant transformation of OE. The study was conducted under the guidelines that had been approved by the medical ethics committee of the Nara Medical University, Kashihara, Japan. Written informed consent was obtained from all patients. Data were acquired under regular clinical care conditions.

Patients who were pathologically diagnosed with OE or their malignant transformation (EAOC) between December 2012 and March 2014 in Nara Medical University Hospital were enrolled in this study. Two patient groups were recruited; 1) pathologically diagnosed benign OE patients within 3 months of the registration (the OE group) and 2) those with previously diagnosed OE with a pathologically proven malignant transformation (the EAOC group). Eligible patients were aged 20 years or older. All EAOC (n = 15) had synchronous endometriotic lesions according to Sampson and Scott’s criteria.^27,28^ A histological examination revealed a transition between the ovarian cancer and the directly adjacent endometriosis. Patients with contraindications to MR imaging (e.g. allergy to all suitable contrast agents, cardiac pacemaker), those with evidence of severe or uncontrolled systemic disease, those with severe psychiatric disorders that affect on informed consent and those with pregnancy or breastfeeding were excluded. Participants were recommended to undergo preoperative (*in vivo*) and/or postoperative (*ex vivo*) MR relaxometry in addition to conventional imaging tests, such as transvaginal ultrasonography, CT and routine MR imaging, performed as part of clinical care. MR relaxometry was scheduled as close as possible to the surgery. Mean (± standard deviation [SD]) interval between MR imaging examination and surgery was 26 ± 29 days (range 0–86 days). According to the above criteria, 82 patients were selected during the period of the study. Sixty-five premenopausal women and two postmenopausal women were selected for the OE group. The EAOC group ultimately consisted of ten premenopausal and five postmenopausal women. Cyst fluid samples were obtained from patients with OE (n = 67) and EAOC (n = 15).

**Fig 1.**
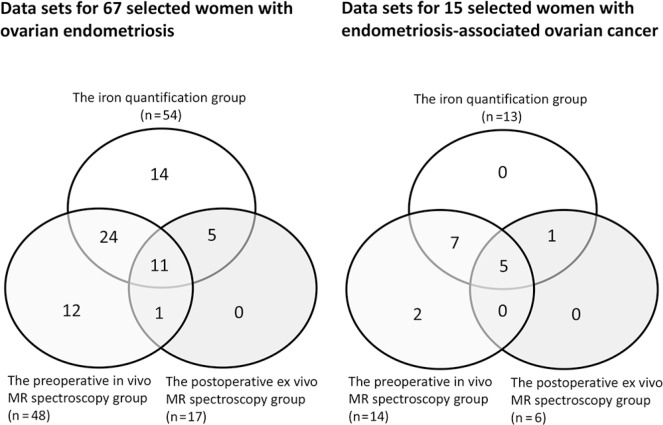
Data sets for 67 selected women with Ovarian endometriosis (OE) and 15 selected patients with Endometriosis-associated ovarian cancer (EAOC). This figure shows distribution of the patients across experimental groups.

Cystic fluids harvested from surgical specimens with a syringe were collected into a plastic tube without an anticoagulant and centrifuged. Supernatants were kept frozen at −20°C within 1 hour of resection until the time of analysis. Samples were used for both biochemical analysis and *ex vivo* MR relaxometry. In some cases, the data were limited, since performing all procedures (quantification of iron levels, pre- and post-operative paired MR relaxometry imaging) was impossible. Among data sets for 67 selected women with OE, 54 (81%), 48 (72%) and 17 (25%) patients underwent in vitro quantification of iron levels, *in vivo* MR relaxometry and *ex vivo* MR relaxometry, respectively (Fig. 1). Among data sets for 15 selected patients with EAOC, there were 13 cases (87%) of the iron quantification group, 14 cases (93%) in the *in vivo* MR relaxometry group and 6 cases (40%) in the *ex vivo* MR relaxometry group (Fig. 1). EAOC patients include different histological subtypes of epithelial ovarian cancer; clear cell carcinoma (n = 9), endometrioid carcinoma (n = 3), mixed-type carcinoma (n = 1), seromucinous carcinoma (n = 1) and undifferentiated carcinoma (n = 1). These tumors were pathologically proven to have arisen from endometriotic epithelial cells. The results of the assays were not available to the clinicians and therefore did not influence subsequent patient management.

### MR relaxometry for determining R2 value

All patients underwent routine MR imaging using T_1_W and T_2_W sequences. MR images were obtained on a 3T system (Magnetom Verio, Siemens Healthcare, Erlangen, Germany). After the routine clinical MR imaging, the registered patients underwent MR relaxometry by using single-voxel acquisition mode sequence at a multiple echo times and by fitting an exponential decay to the echo amplitude at different multiple echo times.^22^ A parameter R2 value (s^−1^) was calculated using high-speed ${\text{T}}_{2}^{*}$ -corrected multi-echo MR sequence (HISTO) by the 3T-MR system *in vivo* and *ex vivo* that has been described previously.^22,29^ The HISTO sequence was based on the single voxel steam sequences that could be used for relative fat quantification in the liver.^30^ This sequence allows estimation of liver iron deposition since T_2_ of water change with iron concentration. The pulse sequence design and programming were done with an imaging platform (Siemens Medical Systems, Erlangen, Germany) and applied to the 3T-system. The sequence used the same minimal TE on 1.5T MR system, reduced to 12 ms by increasing the gradient amplitude between the initial radio frequency pulses. The sequence has fixed number of five measurements with different TE; 12, 24, 36, 48 and 72 ms. The typical protocol is performed in breath hold, with a total acquisition time of 15 sec. The repetition time (TR) was fixed to 3000 ms which proved to be enough to compensate the effects of signal saturation while maintain an acceptable acquisition time. A 15 × 15 × 15-mm spectroscopy voxel (VOI) was placed to select a region encompassing the liquid portion, but not solid portion, of the cyst lumen. The largest cyst fluid was measured if there were any patients who had more than one cyst. The VOI could be located in the center of the OE or EAOC cyst by Dr. J.T. who had more than 15 years of experience in female pelvic MR imaging.

### Quantification of iron concentrations

After the *in vivo* and *ex vivo* MR relaxometry, total iron concentration was quantified in each cyst fluid as described in reference 10. Briefly, cyst fluids were weighed and microwave digested with 50% HNO_3_ and 5% H_2_O_2_. The final sample solution after digestion of each sample was diluted to 0.1 mol/L HNO_3_. The amount of total iron was determined by inductively coupled plasma optical emission spectrometry (ICP-OES) method.

### Preparation of the standard calibration curves

#### Calibration curve of iron working standards

Human oxygenated hemoglobin (oxyHb) was prepared as a standard from blood samples.^31^ Human methemoglobin (metHb) was prepared as a standard from oxyHb by treatment with sodium nitrite.^31^ Furthermore, saccharated ferric oxide was purchased from Nichiikou Inc., (Toyama, Japan). Stock solutions containing human hemoglobins (oxyHb, metHb) and saccharated ferric oxide as a control were prepared in water to mimic the effects of iron found in cyst fluid samples. We diluted the stock solution to produce calibration solutions with various concentrations of saccharated ferric oxide, oxyHb and metHb. Calibration solutions in the 30 ml plastic tubes fixed in agarose phantoms were placed in the MR apparatus and then measured.^32^

#### Calibration curve of *ex vivo* R2 value for iron estimation in cyst fluid samples

The cyst fluid collection comprises 22 samples from patients with OE (n = 16) or EAOC (n = 6) after surgery. The cyst fluid samples in the 30 ml plastic tube were then agar-embedded for *ex vivo* MR relaxometry. The same samples were also used for total iron quantifications by the biochemical ICP-OES method. A calibration curve for *ex vivo* R2 value and total iron concentration was constructed.

#### Calibration curve of *in vivo* R2 value for iron estimation in cyst fluid samples

MR relaxometry was employed for preoperative imaging in patients with OE (n = 35) and EAOC (n = 12). After *in vivo* MR relaxometry measurement, total iron concentrations were determined in the samples collected at the surgery.

#### Correlation between *ex vivo* R2 value and *in vivo* R2 value

We compared the R2 value of the cyst fluid samples (OE, n = 12; EAOC, n = 5) between *in vivo* and *ex vivo* MR relaxometry. A calibration curve for *in vivo* and *ex vivo* R2 value was constructed. Comparison of *in vivo* and *ex vivo* MR relaxometry can examine the effects of individual variability.

### Evaluation of the diagnostic potential of total iron concentration and R2 value for EAOC

The receiver operating characteristics (ROC) curve analysis was conducted to ascertain the utility of total iron concentration and R2 value in discriminating between benign OE and EAOC.

### Statistical analysis

Differences between groups of patients as defined in Fig. 1 were estimated by Mann-Whitney U test. Analyses were performed by SPSS (version 21.0, IBM Corp., Armonk, NY, USA). Multiple linear regression analysis was used to evaluate the contribution of each confounding factor for the R2 value. The ROC curve analysis was used to identify the best discriminating threshold of both parameters for differential diagnosis between EAOC and endometriosis. Statistical significance was assumed at a two-sided *P*-value lower than 0.05.

## Results

### Cyst fluid total iron concentrations

**Fig 2.**
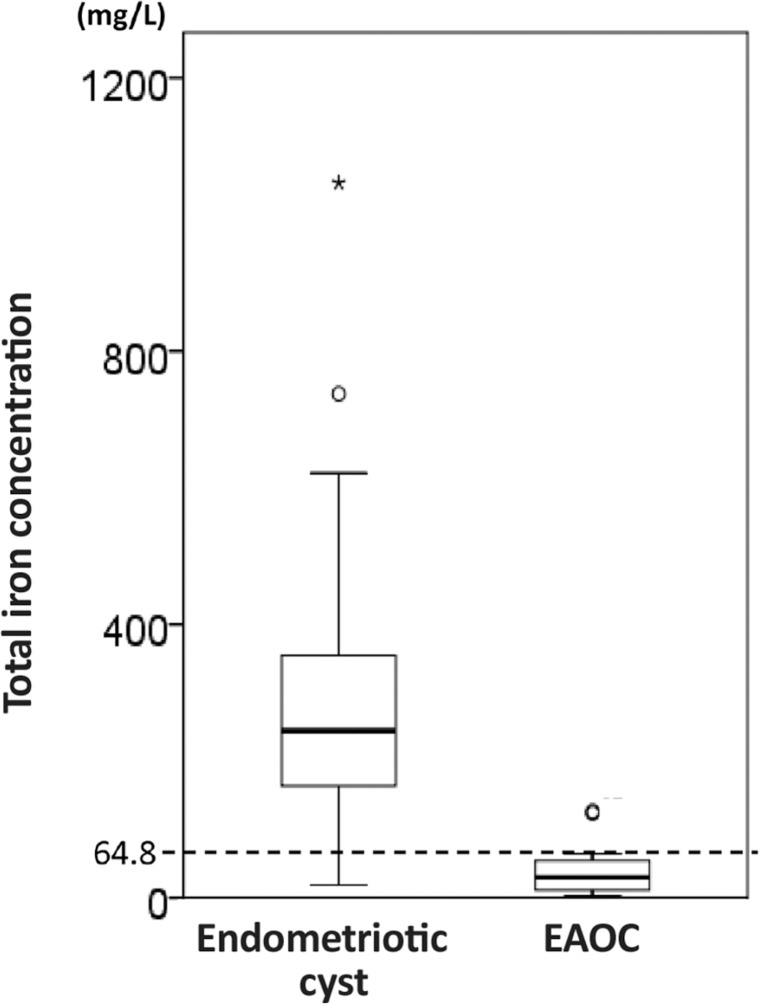
Cyst fluid total iron levels in patients with OE and EAOC. Cyst fluid total iron levels were measured in patients with OE (n = 54) and EAOC (n = 13). This figure shows the distribution of total iron levels for each studied group. EAOC patients showed decreased cyst fluid total iron concentration compared to OE women by biochemical measurement (*P* <0.001).

Patient demographic factors and tumor histology and characteristics have been summarized in Table 1. The coefficient of variation of total iron concentrations determined by biochemical ICP-OES analysis for samples was within 5.0%. Box and whisker plots of the total iron concentrations by cyst type (OE [n = 54] and EAOC [n = 13]) are shown in Fig. 2. Total iron concentration in all samples ranged from 3.0 to 1,046.3 mg/l. The mean (± SD) levels were 302.0 ± 203.2 (range, 65.3 – 1046.3 mg/l) and 33.0 ± 36.6 mg/l (range, 3.0–123.8 mg/l) in OE and EAOC, respectively. Cyst fluid total iron levels were significantly lower in patients with EAOC than in OE (*P* <0.001). The ROC curves were used to compare the power of total iron levels in predicting EAOC from OE. The optimal cutoff point was 64.8 mg/l (sensitivity, 85%; specificity, 98%). Total iron levels were not correlated with age or cyst size (data not shown).

### The validation study

We investigated the potential of *in vivo* R2 value to quantitate the cyst fluid total iron concentration. The validation of the MR relaxometry was achieved in four independent examinations as described below.

#### Phantom experiments: The calibration curve of R2 value and total iron concentration

**Fig 3.**
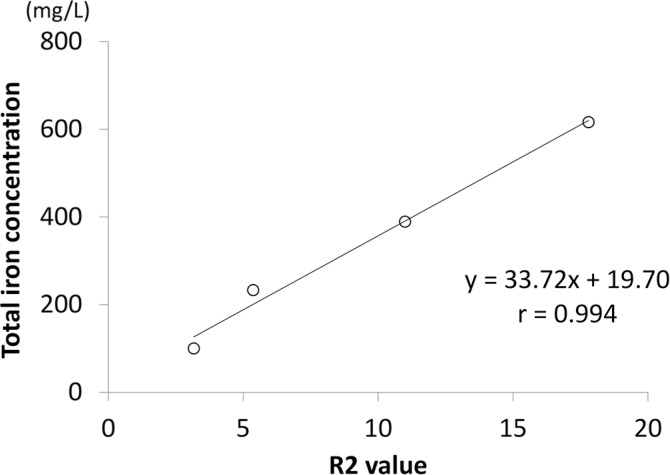
Correlation between the *ex vivo* R2 value and iron working standards. This figure shows the calibration of R2 model for iron estimation. Plastic test tubes were filled with 30 ml of different concentrations of methemoglobin. Calibration solutions in the plastic tubes fixed in agarose phantoms were analyzed by the HISTO sequence applied to the 3T-system. There was a good correlation between R2 value and total iron concentration (r = 0.994). The strong linear correlation between R2 value and iron concentration led to the calibration equation y [total iron] = 33.72 × [*ex vivo* R2] + 19.70. These experiments were repeated twice.

The R2 value showed a positive correlation with iron concentration of metHb (Fig. 3). We also confirmed a linear relationship between the R2 value and saccharated ferric oxide or human oxyHb (data not shown).

#### Correlation between ex vivo R2 value and cyst fluid total iron concentration

**Fig 4.**
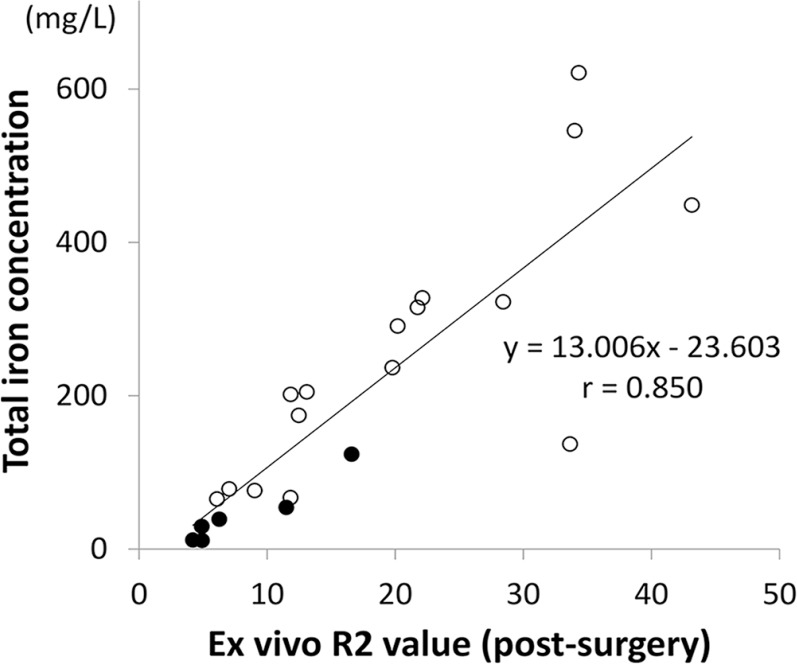
Comparison of the *ex vivo* R2 value and the cyst fluid total iron concentration. Cyst fluid samples (n = 22) were used for both biochemical analysis and *ex vivo* MR spectroscopic imaging scan. Plastic tubes filled with 30 ml of cyst fluids were also measured by the *ex vivo* MR spectroscopy data of a phantom. *Ex vivo* R2 values are shown on the x axes. The y axes represent the total iron concentrations quantitated by the ICP-OES method in OE or EAOC. *Ex vivo* R2 value showed good correlation with the cyst fluid total iron concentration (r = 0.850). ○, OE (n = 16); •, EAOC (n = 6).

*Ex vivo* R2 value showed a strong positive correlation with total iron concentrations in cyst fluid samples [total iron] = 13.006 × [*ex vivo* R2] – 23.603 (r = 0.850) (Fig. 4).

#### Correlation between in vivo R2 value and total iron concentration

**Fig 5.**
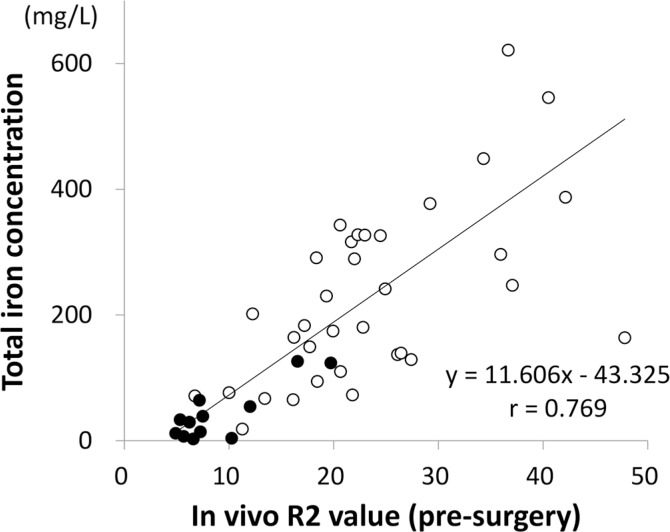
Correlation between *in vivo* R2 value and cyst fluid total iron concentrations. This figure shows the correlation between *in vivo* R2 value and cyst fluid iron concentrations. *In vivo* R2 value showed excellent correlation with the cyst fluid total iron concentration ([total iron] = 11.606 × [*in vivo* R2] – 43.325, r = 0.769). ○, OE (n = 35); •, EAOC (n = 16).

We evaluated the potential correlation between the *in vivo* R2 value of cyst fluid and the quantitation of total iron concentration through the biochemical measurement in 47 surgical samples. Total iron concentration positively correlated with the *in vivo* R2 value (Fig. 5).

#### The relationship between in vivo R2 value and ex vivo R2 value

**Fig 6.**
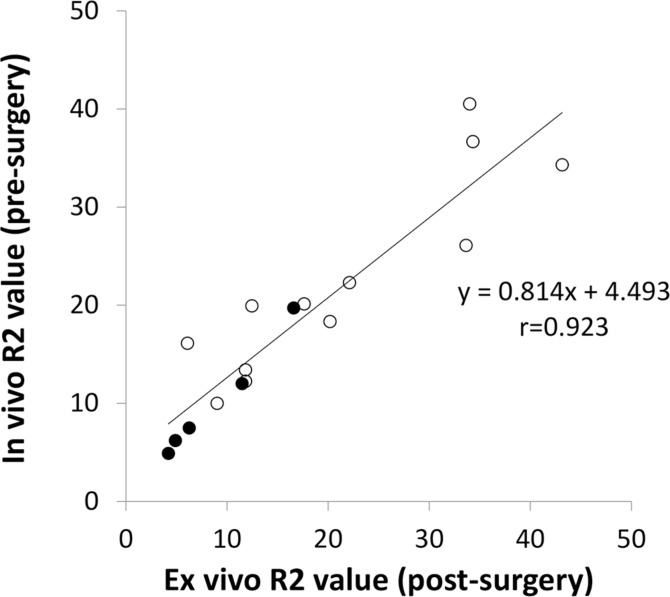
The relationship of cyst fluid R2 value between *in vivo* (pre-surgical) and *ex vivo* (post-surgical) studies. We examined the use of *in vivo* and *ex vivo* R2 values in patients with OE and EAOC. There were significant positive correlations between *in vivo* R2 value and *ex vivo* R2 value. The data were analyzed by linear regression to yield the equations: [*in vivo* R2 value] = 0.814 × [*ex vivo* R2 value] + 4.493. ○, OE (n = 12); •, EAOC (n = 5).

After the measurement of *in vivo* R2 value, cyst fluids were collected. We compared the *in vivo* and *ex vivo* R2 value of 17 cyst fluid samples from the same individuals (OE, n = 12; EAOC, n = 5) (Fig. 6). A positive correlation was observed between *in vivo* and *ex vivo* R2 values (r = 0.923). The *in vivo* R2 value was deduced to be 0.814 from the slope of the *ex vivo* R2 value.

**Fig 7.**
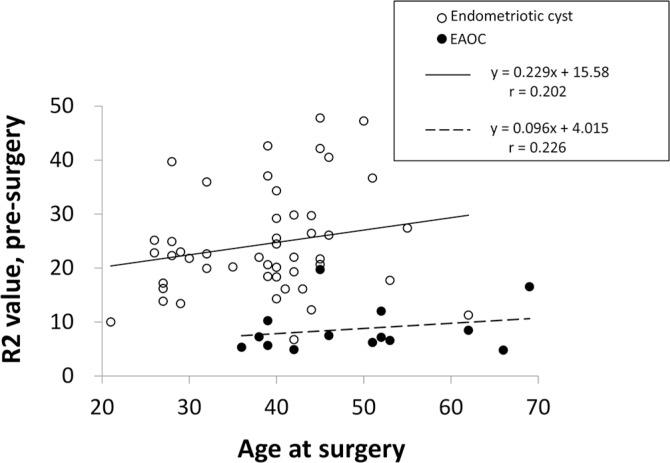
Correlation between cyst fluid R2 value and age at surgery in endometriosis and EAOC. In a subset of 62 patients, comprising endometriosis (open circle, n = 48) and EAOC (closed circle, n = 14), *in vivo* R2 values were measured in cyst fluids. No significant relations between cyst fluid R2 value and age at surgery are seen, exhibiting an adjusted r of 0.202 and 0.226. Compared to OE, EAOC exhibit a decreased level of *in vivo* R2 value, regardless of their age.

**Fig 8.**
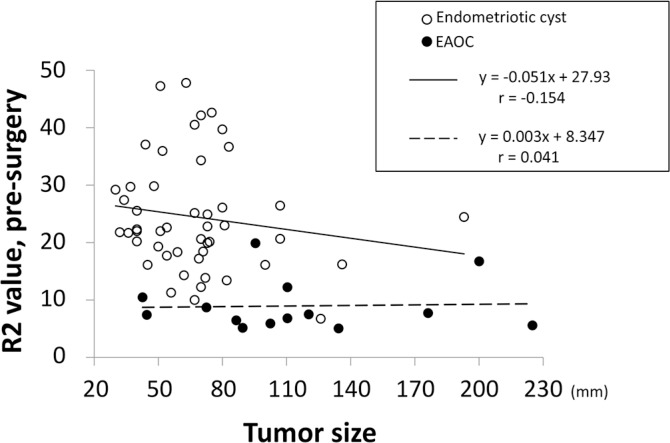
Correlation between cyst fluid R2 value and cyst size in endometriosis and EAOC. In a subset of 62 patients, comprising endometriosis (open circle, n = 48) and EAOC (closed circle, n = 14), *in vivo* R2 values were measured in cyst fluids. No significant relations between cyst fluid R2 value and cyst size are seen, exhibiting an adjusted r of −0.154 and 0.041. Compared to OE, EAOC exhibit a decreased level of *in vivo* R2 value, regardless of their cyst size.

Since the two groups of patients are not homogeneous with respect to the age distribution, we analyzed the correlation between age and R2 value. In analyses of data from OE and EAOC subjects, the *in vivo* R2 value was unrelated to age: [*in vivo* R2 value for OE] = 0.229 × [age] + 15.58, r = 0.202. [*in vivo* R2 value for EAOC] = 0.096 × [age] + 4.015, r = 0.226, *P* = 0.709 (Fig. 7). We did not find any correlation between cyst fluid R2 value and age at surgery. Furthermore, no association of the R2 value with cyst size was also found: [*in vivo* R2 value for OE] = −0.051 × [cyst size] + 27.93, r = −0.154. [*in vivo* R2 value for EAOC] = 0.003 × [cyst size] + 8.347, r = 0.041 (Fig. 8).

### Evaluation of in vivo R2 value as a potential biomarker for differential diagnosis between EAOC and OE

**Fig 9.**
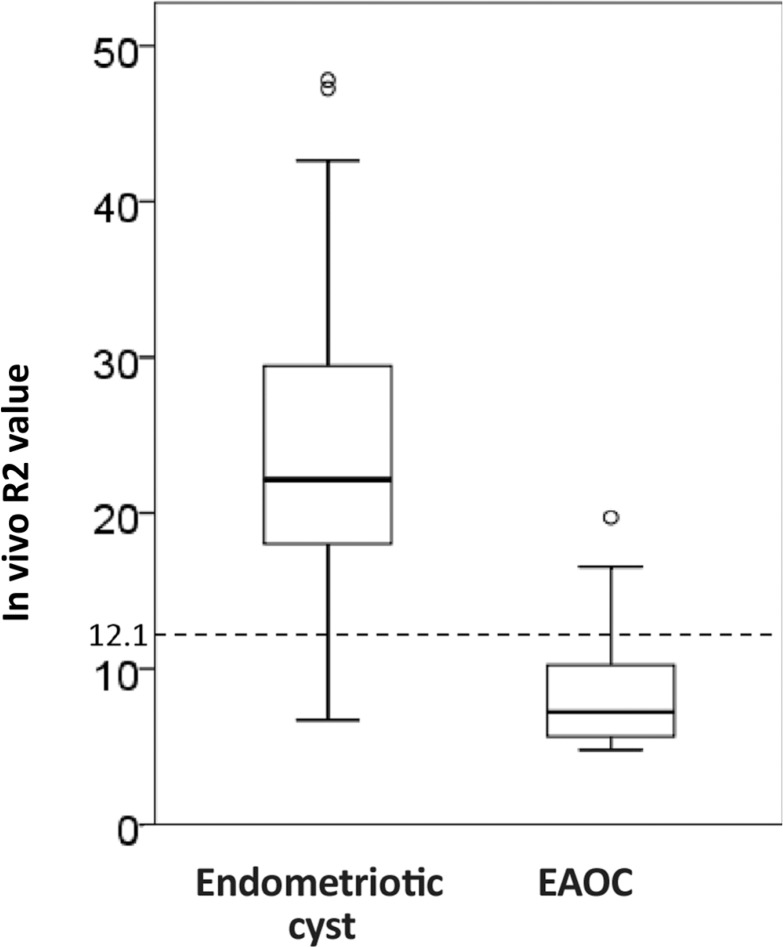
The distribution of cyst fluid *in vivo* R2 value in patients with OE cysts and EAOC. This figure shows the distribution of *in vivo* R2 values for patients with OE (n = 48) and EAOC (n = 14). The dashed horizontal line represents the cut-off level for *in vivo* R2 value. *In vivo* R2 value less than 12.1 was predictive for malignancy.

Box and whisker plots of in vivo R2 value by cyst type (OE, n = 48; EAOC, n = 14) were shown in Fig. 9. The median value and range for R2 value were 22.2 (6.73–47.8) and 7.2 (4.8–19.7) in the OE and EAOC groups, respectively. Mean values ± SD of these groups were also different (24.4 ± 9.8 vs. 8.7 ± 4.5). *In vivo* R2 value was significantly lower in the EAOC group compared with the OE group (*P* < 0.001).

**Fig 10.**
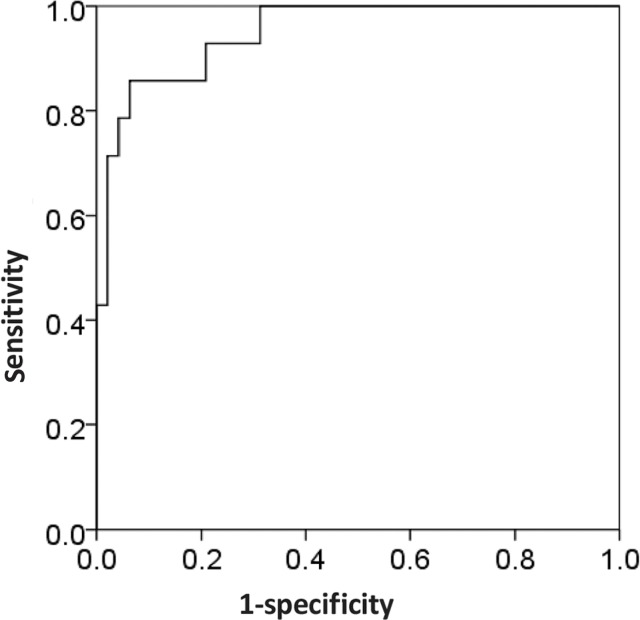
Receiver operating characteristic curves (ROC) of cyst fluid *in vivo* R2 value in patients with OE and EAOC. The best cut off point for malignant transformation of OE established by the ROC curve was 12.1, showing a sensitivity of 86%, specificity of 94%, positive predictive value of 80% and negative predictive value of 96%.

The ROC curve analysis was conducted to ascertain the utility of *in vivo* R2 value in discriminating between OE and EAOC (Fig. 10). The *in vivo* R2 value of 12.1 sec^−1^ yielded a sensitivity of 86%, a specificity of 94%, a positive predictive value of 80% and a negative predictive value of 96% for differential diagnosis of EAOC in benign OE patients.

## Discussion

This series reports the use of MR relaxometry to estimate total iron concentrations in the endometriotic cyst fluid. We compared the theoretical method (*in vivo* and *ex vivo* R2 value) and true iron quantification method (biochemical measurement) to assess the analytical validity of R2 value measurements. The validity of *in vivo* R2 value was supported by *ex vivo* R2 value and total iron concentration data (Figs. 4–6). We found a strong linear correlation between these two methods. A novel equation predicts the cyst fluid total iron concentration (mg of total iron/L of cyst fluid = 11.606 × [*in vivo* R2 value (sec^−1^)] − 43.325). MR relaxometry can potentially be a useful method for non-invasive estimation for total iron concentration. Importantly, no adverse events associated with multi-echo MR relaxometry were reported in any patient.

Next, EAOC patients showed significantly decreased R2 values compared to OE (Fig. 9). A cut-off value of 12.1 to classify patients as OE or having EAOC yielded 86% sensitivity, 94% specificity, 80% positive predictive value and 96% negative predictive value (Fig. 10). Our method provides that *in vivo* R2 value less than 12.1 may be a predictor of malignant transformation. The R2 measurement seems to be a safe, non-invasive and quantitative tool for prediction of malignant transformation of OE. Therefore, *in vivo* R2 value estimation can provide important new information about not only the accuracy of cyst fluid iron quantification, but also differential diagnosis between EAOC and OE.

Routine MR imaging has high sensitivity and specificity for the detection of hemorrhagic content and diagnosis of OE.^33^ The T_2_ shading sign is sensitive for OE.^33^ The presence of an enhancing component within a blood-filled ovarian cyst was considered as suggestive of malignant transformation of a pre-existing OE. The most sensitive MR imaging sign of malignancy in OE is a review of subtraction images.^34^ As described in previous studies,^35,36^ our results also show that EAOC patients have enhanced mural nodules. In addition, a majority of EAOC show large cysts and high or intermediate signal intensity on T_2_-weighted images. Previous studies have demonstrated that the sensitivity, specificity and accuracy for malignancy were 98%, 93%, and 95%, respectively, supporting that contrast-enhanced MR imaging is an accurate method for evaluating the malignancy of adnexal lesions.^37^ Collectively, transverse relaxation rate technology (sensitivity and specificity: 86% and 94%) might be equal to routine MRI as an imaging modality in the assessment of malignancy. Although dynamic contrast-enhanced MR imaging can help to differentiate malignant adnexal masses from benign OE, the diagnostic difficulty still remains in patients without the presence of mural nodules within a cystic mass. Therefore, alternative modalities such as transverse relaxation rate technology are needed. The possibility of a malignant change would be considered if a cyst fluid shows R2 value < 12.1 on transverse relaxation rate technique. However, a need exists for high quality trials with adequate sample sizes to establish clearly the effects of this modality. Future studies will be focused on the potential role of MR relaxometry as a marker for the early detection of borderline malignancy or to enhance our knowledge on a complex, multistep biological process driving OE carcinogenesis.

Evidence suggests that the major pathophysiology associated with OE carcinogenesis is local iron overload.^3^ The homeostatic redox control is achieved by a fine-tuned balance between oxidant and anti-oxidant molecules. Heme and iron overload in OE cysts results in oxidative stress and causes distortion in the redox balance. Oxidant/antioxidant balance function can serve as a double-edged sword, promoting cell death or carcinogenesis.^38,39^ OE patients showed significantly increased R2 values (Fig. 9) or higher iron levels compared to EAOC (Fig. 2). Excess iron-induced oxidative stress could trigger DNA damage and cell death, rather than malignant transformation.^40^ In contrast, patients with EAOC had significantly lower iron levels compared to women with OE. Thus, EAOC might be associated with an effective and optimal antioxidant defense. Upregulation of antioxidant functions in OE cyst may be a molecular event which results in restoration of cell survival, increased chances of accumulation of epigenetic and genetic alterations, and subsequent increase in malignant transformation potential.^38^ The changes of redox balance highlight diverse features involved in hemoglobin, heme and iron homeostasis and the pathogenesis of malignant transformation of OE.

Our study has several limitations. First limitation was the relatively small sample number of patients. Small patient numbers and other factors such as patient age, menopause state, other imaging features may affect our conclusion. Larger cohort studies comparing clinical applicability, discriminative potential, diagnostic accuracy and the predictive values are required to determine in future the optimal test in women with OE or patients with malignant potential. Second, what we actually measured are changes in the spin-spin relaxation time T_2_ (R2 = 1/T_2_) using a single-voxel, multi-echo MR sequence (HISTO). However, changes in T_2_ could be caused by any ferromagnetic substance in the cysts, not only iron. Therefore, we must test for other magnetic substances than iron. Finally, there is a wide range in the time interval between imaging and cyst fluid analysis, which would affect observations related to iron content.

In conclusion, MR relaxometry may be an accurate approach to determine the cyst fluid total iron concentration and represent a non-invasive method to predict malignant transformation of OE. This method might be clinically useful to differentiate EAOC from OE and to select patients for clinical decision making for surgical intervention instead of surveillance.

## Figures and Tables

**Table 1.** Patient demographics and tumor characteristics

Patient and clinical characteristics	Endometriotic cysts	EAOC	*P*-value
number	67	15	
age			
median (range)	39 (21–62)	48 (38–69)	<0.001
mean ± SD	38.9 ± 7.7	49.2 ± 8.8	
premenopause	65	10	0.002
nulliparous	35	8	>0.05
cyst size (mm)*			
median (range)	65 (27–193)	110 (42–225)	<0.001
median (range) interval between *in vivo* MR examination and surgery (day)	1 (0–157)	1 (0–32)	>0.05
median (range) interval between surgery and *ex vivo* MR examination (day)	14.5 (1–83)	22.5 (0–86)	>0.05
FIGO stage	–	Ia (n = 8), Ib (n = 1), Ic (n = 6)	
Pathology	endometriosis	clear cell carcinoma (n = 9) endometrioid carcinoma (n = 3) mixed-type carcinoma (n = 1) seromucinous carcinoma (n = 1) undifferentiated carcinoma (n = 1)	

* maximum diameter of tumors.
